# A ‘Movement Screening Test’ of Functional Control Ability in Female Recreation Golfers and Non-Golfers over the Age of 80 Years: A Reliability Study

**DOI:** 10.3390/jfmk3040054

**Published:** 2018-11-07

**Authors:** Nicholas Webb, Keira Rowsome, Sean Ewings, Mark Comerford, Maria Stokes, Sarah Mottram

**Affiliations:** 1 School of Health Sciences, University of Southampton, Southampton SO17 1BJ, UK; 2 Movement Performance Solutions Ltd., Bristol BS1 3AE, UK; 3 Arthritis Research UK Centre for Sport, Exercise and Osteoarthritis, Nottingham NG7 2UH, UK

**Keywords:** recreational golfers, female, movement screening, aging, functional ability

## Abstract

Assessing function in elderly populations predominantly aims to quantify the risk of falling. Current assessment methods do not consider changes associated with aging in movement coordination patterns and the ability to control movement. The aim of this study was to examine the intra-rater reliability of a ‘Movement Screening Test’ (MST) in females over 80 years across a range of physical activity levels, who were golfers and non-golfers. Female recreational golfers (*N* = 21) and non-golfers (*N* = 10) aged 80 to 87 years performed the MST. The MST consists of three tests: Test 1, sit to stand with arm lift; Test 2, trunk lean with knee bend and opposite arm lift; Test 3, chest rotation with neutral head and pelvis. Videos of the MST were analyzed and scored according to specific criteria. The videos were reviewed on two separate occasions to quantify the intra-rater reliability of scoring of the MST. Intra-rater reliability (κ) of the MST demonstrated substantial agreement for 11/23 criteria (κ = 0.65 and to 0.78) and excellent agreement for 9/23 criteria (κ = 0.81 to 1). Therefore, the reliability of the MST for women aged 80 years and over was established. The MST test and scoring system may be further refined to improve reliability. Further investigations could explore coordination patterns in older people, how these relate to various aspects of musculoskeletal function, and how they vary between different populations.

## 1. Introduction

The World Health Organization suggests that the number of adults over the age of 80 is set to quadruple by the year 2050 [1]. Health expenditure for adults over the age of 75 is approximately 5.5 times higher than for their younger counterparts aged 25–34 in industrialized countries [2].

Much of the health care costs associated with aging are due to the increased risk of falls and associated injuries [3]. The frequency of falling in the older population increases with age with one in three people over the age of 65 and half of adults over 80 falling at least once a year [4]. The reason for falling is often described as multi-factorial [5], though age-related reductions in strength [6,7], balance [8,9,10], and postural stability [9,10], as well as reduced activity levels [11], significantly contribute to falling. The most significant reductions tend to occur in adults over the age of 80 years and have been reported to be more prevalent in women than in men [4,11,12,13]. 

The reduction in activity and subsequent physiological changes in older adults has led to a host of screening tools being developed to measure a number of physical performance characteristics. The Senior Fitness Test [14], Timed up and Go (TUG) Test [15], Berg Balance Test [16], and the Functional Reach Test [17] are just a few of many tests that are used to assess the physical performance of older adults. However, these quantitative measures do not focus on movement patterns that influence the scores, and so do not provide health professionals with specific information regarding rehabilitation strategies.

These tests are also predominantly used to identify potential falls risks in older adults and, whilst they aim to reflect functional performance, they do not assess the ability of individuals to coordinate their movements.

Movement is clearly complex and influenced by many interactions. An adapted model of the dynamic systems theory has been presented by Dingenen [18]. This model proposes that an individual’s movement pattern emerges out of the interaction of three domains. These domains include factors related to the person (e.g., age, co-morbidities), the task being performed (e.g., sitting to standing, walking), and the environment or context in which it is performed (walking within the confines of home or outdoors). Clinical interventions can focus on any of these domains in order to produce a clinical outcome. However, a focus upon the movement pattern emerging from these interactions is of interest to both clinicians and researchers [18]. The value of movement and the factors that influence movement coordination strategies are now being used to support participation and maintain health in the older population [19].

An ability to consciously demonstrate variation in the co-ordination strategies employed to achieve a movement outcome can be considered to illustrate the possession of choice in movement [18,20]. For example, when transitioning from sitting to standing, an individual may choose to employ a movement strategy that is reliant upon a large amplitude of sagittal plane movement at the hip, whereas in their next demonstration of the same action, they employ a strategy in which there is approximately equal contribution from the hip and knee. The capacity to alter at will which, of all the available strategies, is employed is proposed to act as a marker of the health of the individual’s movement system [18,20]. Cognitive movement control assessment evaluates an individual’s ability to cognitively coordinate movement at a specific joint or region (site) in a particular plane of movement (direction), under low- and high-threshold loading, often during multi-joint tests within functionally orientated tasks [21,22].

These tests have demonstrated good to excellent inter- and intra-rater reliability [22,23]. Adopting this movement coordination approach to screen older adults for loss of choices in movement, may aid in targeting rehabilitation programs and help promote the importance of maintaining activity levels in older populations. Some evidence is emerging of the benefits of motor learning exercises on mobility performance in older adults [24,25,26].

A battery of cognitive movement control tests, designed for older people, was described by Rowsome et al. [23]. Overall, intra-rater reliability for a group of participants aged 65–79 years was acceptable, but certain assessment criteria were identified as being less reliable than others. Recommendations were made for the refinement of some criteria to improve the reliability of the screening tool, so the present study examined the reliability of a refined tool in older people over 80 years. Refining this movement screen to identify coordination strategies in older adults may aid in targeted rehabilitation programs and help promote the importance of maintaining movement choices in older populations.

The primary aim of this study was to determine the intra-rater reliability of a Movement Screening Test (MST) in golfers and non-golfers females aged over 80 years, across a range of physical activity levels.

## 2. Materials and Methods

### 2.1. Participants

Thirty-one participants were included in the study, 10 non-golfers and 21 golfers. Golfers were recruited by contacting local golf clubs in Hampshire and West Sussex and displaying information about the study. To recruit non-golfing participants, posters were sent, and short presentations were delivered to groups at local churches, luncheon clubs, and libraries. Fifty potential participants were contacted: 11 non-golfers and 8 golfers did not meet the inclusion and exclusion criteria. The study was approved by the Faculty of Health Sciences ethics committee, University of Southampton, ethics number 14,158. All procedures performed were in accordance with the ethical standards of the institutional research committee and with the 1964 Helsinki declaration and its later amendments.

In order to be deemed suitable for the study, all participants answered a screening questionnaire which included a section adapted from the Physical Activity Scale for the Elderly (PASE) [27] to ensure the non-golfing participants did not regularly partake in moderate levels of physical activity. Examples of moderate activities given in the PASE were golf without a cart, ballroom dancing, doubles tennis. Golfers were included if they had been playing golf for more than two years and regularly played 18 holes of golf at least once per week.

All participants were screened over the phone prior to participation in the study. Participants were excluded if they had: musculoskeletal disorders that limited their daily activities; joint replacement in the previous five years; cardio respiratory disorders including history of myocardial infarction; uncontrolled blood pressure; chronic obstructive pulmonary disease; systemic disorders such as uncontrolled diabetes; history of malignancy within the previous five years; neurological conditions such as Parkinson’s, Multiple Sclerosis, and Rheumatoid Arthritis. If known, height and weight were asked to determine the body mass index (BMI). A BMI > 35kg/m^2^ excluded participants from the study.

### 2.2. Procedure

The testing procedures were carried out at either a local golf club or at the University of Southampton, and the testing procedures included in the present study were completed in 20 min. Prior to data collection, all participants were asked to refrain from any strenuous physical activity in the 48 h prior to testing. On arrival, all participants completed a consent form. Prior to testing, all participants’ height and body mass were recorded.

### 2.3. Movement Screening Tests

**Figure 1:**
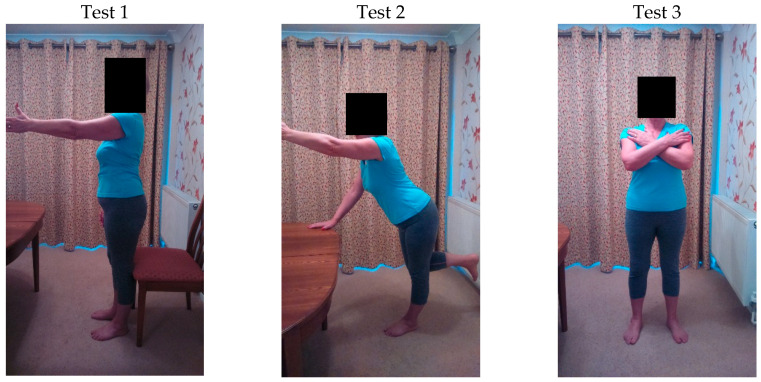
Participant carrying out the Movement Screening Tests: Test 1, sit to stand with arm lift; Test 2, trunk lean with knee bend and opposite arm lift; Test 3, chest rotation with neutral head and pelvis.

All participants completed the movement screening tests outlined in Appendix A. Figure 1 shows the three tests being performed. The participants were videoed using a handheld recording device (Samsung Galaxy Nexus) for offline assessment of the test. Test 1 was performed twice by each participant, allowing the test to be recorded in the frontal (dominant side) and sagittal planes. Test 2 was recorded in the frontal plane, allowing the dominant arm to be viewed. Test 3 was recorded in the sagittal plane. Prior to commencing the tests, the researcher explained in detail the objective of each test, and the participants were shown videos of each test being performed. The participants were allowed up to two practice attempts so that they understood how to perform the movements. Specific standardized instructions were then given verbally whilst the participant performed each test.

### 2.4. Scoring System

After all participants had completed the MST, the videos were reviewed and scored according to a scoring system (see Appendix A). This scoring system was adapted with permission from the original MST [23]. The tests were then reviewed and scored again two weeks later to allow intra-rater reliability to be assessed. As the test is designed to measure impairment, a score of 0 is rated a pass, and a fail is rated as 1. The participants were scored for each individual test, and a total score out of 23 points was also calculated.

### 2.5. Statistical Analysis

All data were analyzed using IBM SPSS (version 22, SPSS Inc., Chicago, IL, USA), Microsoft Excel, and Stata and were initially tested for normality using the Shapiro–Wilks test.

Movement Screening Test: Intra-rater reliability. Cohen’s Kappa coefficient (κ) including 95% confidence intervals and percentage agreement was used to establish the reliability of each individual test criterion for the MST. Kappa is commonly used when ratings are on a nominal scale [28] and describes the agreement beyond chance, whereas percentage agreement does not take this into account. According to McHugh [29], Kappa value ranges between 0.41 and 0.6 are considered moderate, between 0.61 and 0.8 are considered substantial, and between 0.81 and 1 are considered excellent.

The reliability of the overall movement screen test scores was assessed using the intra-class correlation co-efficient (ICC) [30]. The ICC (1, 1) model was used (with participants as the only effect). According to Fleiss [30], ICC values >0.75 are considered excellent, between 0.4 and 0.74 good to fair, and <0.4 poor.

## 3. Results

### 3.1. Participant Characteristics

Participant characteristics for the golfers and non-golfers are presented in Table 1. No significant differences were found for height, weight, and BMI (*p* > 0.05) between the two groups. A significant difference for age (*p* < 0.05) was observed, with the golfers being older than the non-golfers. Results from the screening questionnaire described the activity levels of the golfers and non-golfers. Of the 21 golfers (who averaged two rounds per week, and five of whom played three times per week) one also took part in 1–2 days of strenuous activity per week and all 21 took part in moderate activity each week (mean 2.5 days per week) with two taking part in four days of moderate activity per week; in addition, ten took part in other forms of light recreational activity in addition to golf, with a mean of approximately 2.5 days per week. As for non-golfers, none of them took part in any moderate or strenuous activity; of the 10 non-golfers, five took part in light recreational activity each week (three participants took part on three days in any week, two on just one day in any week).

#### Percentage Agreements

**Figure 2:**
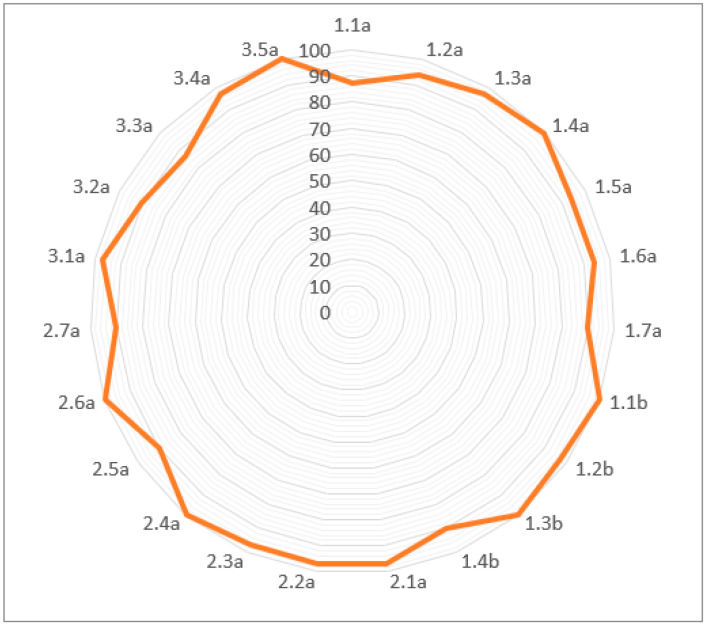
Radar graph showing the percentage agreement for individual test criteria (%).

The percentage agreements for individual test criterions ranged from 87 to 100%, with a mean overall agreement of 95%. Only two of the 23 criteria fell below 90% (Test 1.1a and 3.3). Six criteria (Test 1.4a, 1.1b, 1.3b, 2.4, 2.6, 3.5) demonstrated 100% agreement. The percentage agreements for each test are shown in Table 2. A graphical representation in Figure 2 demonstrates clearly the percentage agreement for each of the test criteria, with values close to or on the outer edge of the graph representing excellent agreement.

### 3.2. Kappa Values

Kappa values for each test criterion ranged from 0.47 to 1. Only one of the 23 screening criteria (test 3.4) demonstrated kappa values below 0.6 (0.47), demonstrating moderate agreement. However, 97% agreement was observed for this test. Eleven of the 23 criteria demonstrated kappa values between 0.65 and 0.78, indicating substantial agreement. Nine criteria reported kappa values between 0.81 and 1, which were considered excellent. Kappa values for two criteria, test 2.6 and 3.5, were not reported (NA), as no fail (1) scores were reported for any of the participants. 

All test criteria percentage agreements and Kappa values with 95% confidence intervals are presented in Table 2.

The intra-class correlation coefficient (ICC 1, 1) of the overall scores (total of the 23 criteria) was 0.74 with 95% confidence intervals, 0.53–0.86. This demonstrates good agreement of the overall test score. 

## 4. Discussion

The aging process has detrimental effects on our physiology and can fundamentally alter the way we move. Assessing movement choices in the elderly population may help direct prevention strategies and rehabilitation interventions to improve the physical function and prevent falls.

The intra-rater reliability (*k*) of the MST demonstrated substantial to excellent agreement for all but one criteria (test 3.4). This is in line with other tests that aim to quantify functional ability in older populations with similar reliability values reported for the TUG, Berg Balance Scale, and Gait speeds [31].

To allow for observation of the movement patterns two weeks apart, observations were made from video recordings. This method of quantifying movement has proved problematic in other studies [22], as the camera set up does not allow freedom to assess all angles and distances. Mischiati et al. [22] also highlighted similar problems as those experienced in the present study, in that the ability to stabilize the body may take a few seconds when performing dynamic movements, and thus the scoring should reflect this. For example, in Test 2, the participants were asked to lift their hand and opposite foot. Reducing the base of support challenges the movement outcome. However, in some cases, this destabilization caused a momentary period of loss of control, though the participants were able to gain postural control after a few seconds. Therefore, the instruction on when to score the movement may need further clarification. Scoring the movement after a pre-determined time, for example, 3 s, would help to standardize the scoring. This should be considered for future studies when determining inter-rater reliability.

This study illustrates situations where it is not possible to assess agreement using Kappa (κ). This phenomenon of high agreement but low Kappa scores was discussed by Cicchetti and Feinstein [32]. In the present study, for example, the scores for each participant were “0” in tests 2.6 and 3.5, for which no fail (1) scores were reported for any of the participants. κ was therefore undefined; this is because there were no data relating to “1”, and so the agreement could not be assessed across all out-comes (0 and 1). In such cases, κ was presented as “N/A”. Both these tests had 100% agreement on one level of the outcome, and no information regarding agreement on the other level of the outcome.

Two movement screening reliability studies report on intra-tester reliability using cognitive movement control tests. Mischiati et al. [22] concluded that both inter- and intra-rater reliability of tests in The Foundation Matrix are acceptable when rated by two experienced therapists. Overall, the test percentage agreement was 87% for inter-rater reliability, with 98% Rater 1, 94% Rater 2 for test re-test reliability. ICCs were excellent within raters (Rater 1, 0.96; Rater 2, 0.88). The reliability for individual components of each test was more variable: intra-rater, 88–100% Rater 1, 75–100% Rater 2. Cohen’s Kappa values for intra-rater were 0.6–1.0 for Rater 1, −0.1–1.0 for Rater 2. Rowsome et al. [23] reported on intra-rater reliability in a similar battery of tests on thirty-one female recreational golfers, aged 65–77. The percentage agreement for each test ranged from 93.0 to 97.3%, with an overall mean agreement of 95.5%. The Kappa values for the test scores ranged from 0.35 to 0.90. the percentage agreement for individual criteria ranged from 83.0 to 100.0%, with kappa values ranging from 0.00 to 1.00.

Changes were made to aspects of how the tests maneuvers were conducted and to the assessment criteria. Examples include: (i) amendments were made to score movement challenges with the dominant side only (direct comparison to the Rowsome study could not be made) (ii) changing the assessment criteria of test 3.1 from ‘can they keep the head facing forward and control/prevent the head from turning or rotating to follow the upper trunk rotation? ’ to ‘can they keep the head facing forward and control/prevent the head from turning or rotating throughout the 30° of upper trunk rotation?’ Rowsome et al. [23] reported 90.3% agreement, whereas this paper determined a 97% agreement, demonstrating refinement of the MST for question 3.1.

### Limitations and Future Recommendations

The ability to recruit older adults has also been shown to be problematic. Crombie et al. [11] demonstrated that females over the age of 75 years are reluctant to participate in research projects. Therefore, it may be that the participants recruited in this study were not typically representative of a female population over the age of 80 years. When comparing this age group with those of other studies [33], participants with a wide range of health complications were included by others, suggesting this feature is representative of this population. For future studies, it may be prudent to consider relaxing the inclusion and exclusion criteria to allow comparisons of groups that are more representative of an aging population; for example, by including those with joint replacements.

Now the reliability of the MST has been established, studies are warranted to test the validity of the MST against motion analysis, discriminant validity (to distinguish between groups), and sensitivity to change. Further research is needed to explore the relationship between the observed loss of movement choice, notated by the site, direction and threshold of uncontrolled movement, and other measures of musculoskeletal function.

## 5. Conclusions

The reliability of a movement screening test in female golfers and non-golfers over the age of 80 years has been established. The results illustrate the utility of the MST for observing coordination strategies and the ability to control movement in older people, which has been lacking in assessments for older people. Identifying variations in coordination strategies using the MST may aid in targeting rehabilitation programs to support participation and maintain health in the older population.

## Figures and Tables

**Table 1:** Participant characteristics: Age is presented as median (interquartile range). Height, weight, and body mass index (BMI) are presented as mean ± SD with mean difference (confidence intervals).

Characteristics	Golf (*N* = 21)	Non-Golf (*N* = 10)	Mean Difference (95% CI)	Significance
Age (Years) Range	83 (3) 80–87	80.5 (1.25) 80–83	N/A	*p* = 0.004 *
Height (m) Range	1.58 ± 0.04 1.51–1.65	1.57 ± 0.05 1.46–1.66	−0.01 (−0.05–0.02)	*p* = 0.38
Weight (kg) Range	62.3 ± 9.22 46.9–78.8	66.2 ± 12.7 53.4–93.8	3.87 (−4.34–12)	*p* = 0.34
BMI (kg/m^2^) Range	24.7 ± 3.56 18.8–31.9	26.8 ± 4.42 20.7–34	2.05 (−0.97–5.0)	*p* = 0.34

* Denotes significance *p* < 0.05.

**Table 2:** Reliability of individual test scores; % agreements and Cohen’s kappa with 95% confidence intervals.

Test	% Agreement	Kappa
1.1a	87	0.74 (0.50–0.97)
1.2a	94	0.71 (0.33–1.00)
1.3a	97	0.65 (0.02–1.00)
1.4a	100	1 (1.00–1.00)
1.5a	94	0.85 (0.65–1.00)
1.6a	94	0.81 (0.57–1.00)
1.7a	90	0.73 (0.45–1.00)
1.1b	100	1 (1.00–1.00)
1.2b	97	0.78 (0.37–1.00)
1.3b	100	1 (1.00–1.00)
1.4b	90	0.87 (0.69–1.00)
2.1	97	0.87 (0.69–1.00)
2.2	97	0.65 (0.02–1.00)
2.3	97	0.65 (0.02–1.00)
2.4	100	1 (1.00–1.00)
2.5	90	0.73 (0.49–0.97)
2.6	100	NA
2.7	90	0.66 (0.32–1.00)
3.1	97	0.93 (0.81–1.00)
3.2	90	0.67 (0.34–1.00)
3.3	87	0.73 (0.49–0.97)
3.4	97	0.47 (−0.12–1.00)
3.5	100	NA

## Appendix A. Movement Screen Test Instructions and Scoring System

**Table A1:** Movement Screen Test Instructions and Scoring System.

Test Details	Scoring Criteria
**Test 1a:** Sit to stand with arm lift (Side View) Sitting with feet on the floor and spine straight, lean 30° forward at the hips. Then stand up keeping the knees out over the feet. Then, maintaining a neutral scapular position, lift the dominant arm to 90° shoulder flexion and lower back to the side.	1: Can they keep the upper back straight and the collar bones up (the chest should not curl into flexion) during: the forward lean to a standing position? the horizontal arm lift and lower? 2: Can they keep the shoulders level and steady (prevent the shoulder hitching into elevation) during the horizontal arm lift? 3: Can they keep the shoulders level and steady (preventing the shoulder dropping down from the neutral position) during the arm lowering action? 4: Can they keep the thumb pointing towards the ceiling to control/prevent the shoulder from turning into medial rotation as the arm is lifted to horizontal? (the palm should be facing inwards) 5: Can they keep the low back straight (not rounding into flexion) during the forward trunk lean? 6: Can they keep the low back straight (not arching into extension) during: -the forward trunk lean? -the movement from sitting to standing? 7: Can they fully straighten at the hips when they stand up? (There should be a vertical line from the trunk through the legs. The trunk should not be leaning forwards or the hips pushed backwards) Total Score out of 7.
**Test 1b:** Sit to stand with arm lift (Front View)	1: Can they keep the shoulders level and steady (control/prevent the shoulder blade hitching into elevation) during the horizontal arm lift? 2: Can they keep the shoulders level and steady (control/prevent the shoulder blade from dropping down from the neutral position) into depression during the arm lowering action? 3: Can they keep the thumb pointing towards the ceiling to control/prevent the shoulder from turning into medial rotation as the arm lifts to horizontal? (the palm should be facing inwards) 4: Can they keep the knees apart (over the feet, preventing the knees from collapsing in towards each other) during the sit-to-stand movement? Total Score out of 4.
**Test 2:** Trunk lean with knee bend and opposite arm lift. With hand support on a bench/table perform 45° forward trunk lean (independent hip flexion) + knee flexion to lift non-dominant foot off the floor and opposite arm lift to horizontal shoulder flexion.	1: Can they control/prevent the head from dropping forward or lifting from their natural position during: -the trunk forward lean? -arm lifting and lowering? 2: Can they keep the shoulder and trunk steady on the weight-bearing arm? 3: Can they keep the non-weighting shoulder steady as the arm is lifted and reaches out? 4: Can they keep the thumb pointing towards the ceiling as the arm lifts to horizontal? (the palm should be facing inwards). 5: Can they keep the back straight and not let it round out into flexion during the forward lean? 6: Can they keep the back straight and not let it arch into extension? 7: Can they keep the pelvis from rotating (stay facing the front) during: -the foot lift? -the opposite arm lift? Total Score out of 7.
**Test 3:** Chest rotation with neutral head and pelvis. The range of upper body rotation should be 30° (using a line across the tips of the shoulders) towards the dominant side, without any head, pelvic, or leg movement Participants will perform a small knee bend and then rotate their trunk to 30° towards their dominant side.	1: Can they keep the head facing forward and control/prevent the head from turning or rotating throughout the 30° of upper trunk rotation? 2: Can they keep their shoulders level and in a neutral mid position (prevent hitching into elevation) during trunk rotation? 3: Can they keep the pelvis facing the front and not allow it to rotate to follow the upper body (no rotation of the pelvis)? 4: Can they keep the knees apart and stationary during the chest turn? (no inward or outward movement of the knees across the line of the feet) 5: Can they keep the feet facing forward with the inside edges of the feet parallel and prevent the foot from turning in or out during the chest turn? Total Score out of 5.
Total of all test scores out of 23.	
